# Smoothing Strategies Combined with ARIMA and Neural Networks to Improve the Forecasting of Traffic Accidents

**DOI:** 10.1155/2014/152375

**Published:** 2014-08-28

**Authors:** Lida Barba, Nibaldo Rodríguez, Cecilia Montt

**Affiliations:** ^1^Pontificia Universidad Católica de Valparaíso, 2362807 Valparaíso, Chile; ^2^Universidad Nacional de Chimborazo, 33730880 Riobamba, Ecuador

## Abstract

Two smoothing strategies combined with autoregressive integrated moving average (ARIMA) and autoregressive neural networks (ANNs) models to improve the forecasting of time series are presented. The strategy of forecasting is implemented using two stages. In the first stage the time series is smoothed using either, 3-point moving average smoothing, or singular value Decomposition of the Hankel matrix (HSVD). In the second stage, an ARIMA model and two ANNs for one-step-ahead time series forecasting are used. The coefficients of the first ANN are estimated through the particle swarm optimization (PSO) learning algorithm, while the coefficients of the second ANN are estimated with the resilient backpropagation (RPROP) learning algorithm. The proposed models are evaluated using a weekly time series of traffic accidents of Valparaíso, Chilean region, from 2003 to 2012. The best result is given by the combination HSVD-ARIMA, with a MAPE of 0 : 26%, followed by MA-ARIMA with a MAPE of 1 : 12%; the worst result is given by the MA-ANN based on PSO with a MAPE of 15 : 51%.

## 1. Introduction

The traffic accidents occurrence is a matter of impact in the society, therefore a problem of priority public attention; the Chilean National Traffic Safety Commission (CONASET) periodically reports a high rate of sinister on roads; in Valparaíso from year 2003 to 2012 28595 injured people were registered. The accuracy in the projections enables the intervention by the government agencies in terms of prevention; another demandant of information is the insurance companies, who require this kind of information to determine new market policies.

In order to capture the dynamic of traffic accidents, during the last years some techniques have been applied. For classification, decision rules and trees [1, 2], latent class clustering and bayesian networks [3], and the genetic algorithm [4] have been implemented. For traffic accidents forecasting, autoregressive moving average (ARMA) and ARIMA models [5], state-space models [6, 7], extrapolation [8], dynamic harmonic regression combined with ARIMA, and dynamic transfer functions [9] have been implemented.

The smoothing strategies Moving Average (MA) and Singular Value Decomposition (SVD) have been used to identify the components in a time series. MA is used to extract the trend [10], while SVD extracts more components [11]; the SVD application is multivariate and in some works is applied for parameter calibration in dynamical systems [12, 13], in time series classification [14], or to switched linear systems [15]; typically SVD has been applied over an input data set to reduce the data dimensionality [16] or to noise reduction [17].

ARIMA is a linear conventional model for nonstationary time series; by differentiation the nonstationary time series is transformed in stationary; it is based on past values of the series and on the previous error terms for forecasting. ARIMA has been applied widely to model nonstationary data; some applications are the traffic noise [18], the daily global solar radiation [19], premonsoon rainfall data for western India [20], and aerosols over the Gangetic Himalayan region [21].

The autoregressive neural network (ANN) is a nonlinear method for forecasting that has been shown to be efficient in solving problems of different fields; the capability of learning of the ANN is determined by the algorithm. Particle swarm optimization (PSO) is a population algorithm that has been found to be optimal; it is based on the behaviour of a swarm; this is applied to update the connections weights of the ANN; some modifications of PSO have been evaluated based on variants of the acceleration coefficients [22], others apply the adaptation of the inertia weight [23–26], also the usage of adaptive mechanisms for both inertia weight and the acceleration coefficients based on the behaviour of the particle at each iteration have been used [27, 28]. The combination of ANN-PSO has improved the forecasting over some classical algorithms like backpropagation (BP) [29–31] and least mean square (LMS) [32]. Another learning algorithm that has been shown to be better than backpropagation is RPROP and is also analyzed by its robustness, easy implementation, and fast convergence regarding the conventional BP [33, 34].

The linear and nonlinear models may be inadequate in some forecasting problems; consequently they are not considered universal models; then the combination of linear and nonlinear models could capture different forms of relationships in the time series data. The Zhang hybrid methodology that combines both ARIMA and ANN models is an effective way to improve forecasting accuracy; ARIMA model is used to analyze the linear part of the problem and the ANN models, the residuals from the ARIMA model [35]; this model has been applied for demand forecasting [36]; however some researchers believe that some assumptions of Zhang can degenerate hybrid methodology when opposite situation occurs; Kashei proposes a methodology that combines the linear and nonlinear models which has no assumptions of traditional Zhang hybrid linear and nonlinear models in order to yield the more general and the more accurate forecasting model [37].

Based on the arguments presented in this work, two smoothing strategies to potentiate the preprocessing stage of time series forecasting are proposed; 3-point MA and HSVD are used to smooth the time series; the smoothed values are forecasted with three models; the first is based on ARIMA model, the second in ANN is based on PSO, and the third in ANN is based on RPROP. The models are evaluated using the time series of injured people in traffic accidents occurring in Valparaíso, Chilean region, from 2003 to 2012 with 531 weekly registers. The smoothing strategies and the forecasting models are combined and six models are obtained and compared to determine the model that gives the major accuracy. The paper is structured as follows.  Section 2 describes the smoothing strategies.  Section 3 explains the proposed forecasting models.  Section 4 presents the forecasting accuracy metrics.  Section 5 presents the results and discussions. The conclusions are shown in Section 6.

## 2. Smoothing Strategies

### 2.1. Moving Average

**Figure 1:**
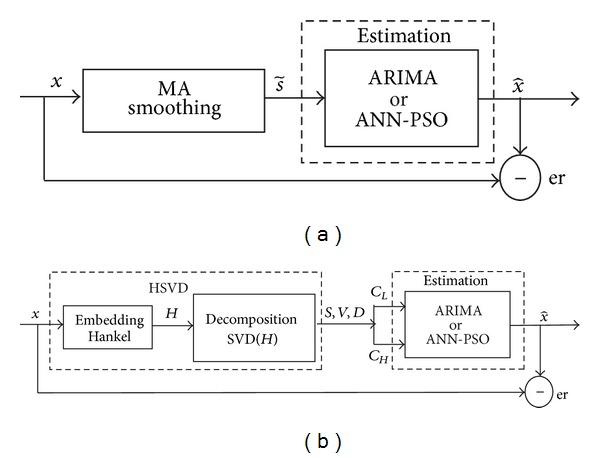
Smoothing strategies: (a) moving average and (b) Hankel singular value decomposition.

Moving average is a smoothing strategy used in linear filtering to identify or extract the trend from a time series. MA is a mean of a constant number of observations that can be used to describe a series that does not exhibit a trend [38]. When 3-point MA is applied over a time series of length *n*, the *n* − 2 elements of the smoothed series are computed with

(1) $$\begin{array}{c} {\tilde{s}}_{k}=\overset{k+1}{\underset{i=k-1}{\sum }}\frac{x_{i}}{3}, \end{array}$$

where ${\tilde{s}}_{k}$ is the *k*th smoothed signal element, for *k* = 2,…, *n* − 1,  *x*_*i*_ is each observed element of original time series, and terms ${\tilde{s}}_{1}$ and ${\tilde{s}}_{n}$ have the same values of *x*_1_ and *x*_*n*_, respectively. The smoothed values given by 3-points MA will be used by the estimation process through the selected technique (ARIMA or ANN); this strategy is illustrated in Figure 1(a).

### 2.2. Hankel Singular Value Decomposition

The proposed strategy HSVD is implemented during the preprocessing stage in two steps, embedding and decomposition. The time series is embedded in a trajectory matrix; then the structure of the Hankel matrix is applied, the decomposition process extracts the components of low and high frequency of the mentioned matrix by means of SVD, the smoothed values given by HSVD are used by the estimation process, and this strategy is illustrated in Figure 1(b).

The original time series is represented with *x*, *H* is the Hankel matrix, *U*, *S*, *V* are the elements obtained with SVD and will be detailed more ahead, *C*_*L*_ is the component of low frequency, *C*_*H*_ is the component of high frequency, $\hat{x}$ is the forecasted time series, and *er* is the error computed between *x* and $\hat{x}$ with

(2) $$\begin{array}{c} \hat{e}r=x-\hat{x}. \end{array}$$

#### 2.2.1. Embedding the Time Series

The embedding process is illustrated as follows:

(3) $$\begin{array}{c} H_{M\times L}=\left[\begin{array}{cccc} x_{1} & x_{2} & \cdots & x_{L}\\ x_{2} & x_{3} & \cdots & x_{L+1}\\ \vdots & \vdots & \vdots & \vdots \\ x_{M} & x_{M+1} & \cdots & x_{n} \end{array}\right], \end{array}$$

where *H* is a real matrix, whose structure is the Hankel matrix, *x*_1_,…, *x*_*n*_ are the original values of the time series, *M* is the number of rows of *H* and also *M* is the number of components that will be obtained with SVD, *L* is the number of columns of *H*, and *n* is the length of the time series. The value of *L* is computed with

(4) $$\begin{array}{c} L=n-M-1. \end{array}$$

#### 2.2.2. Singular Value Decomposition

The SVD process is implemented over the matrix *H* obtained in the last subsection. Let *H* be an *M* × *N* real matrix; then there exist an *M* × *M* orthogonal matrix *U*, an *N* × *N* orthogonal matrix *V*, and an *M* × *N* diagonal matrix *S* with diagonal entries *s*_1_ ≥ *s*_2_ ≥ ⋯≥*s*_*p*_, with *p* = min⁡⁡(*M*, *N*), such that *U* ^*T*^ *HV* = *S*. Moreover, the numbers *s*_1_, *s*_2_,…, *s*_*p*_ are uniquely determined by *H* [39]:

(5) $$\begin{array}{c} H=U\times S\times V^{T}. \end{array}$$

The extraction of the components is developed through the singular values *s*_*i*_, the orthogonal matrix *U*, and the orthogonal matrix *V*, for each singular value is obtained one matrix *A*_*i*_, with *i* = 1,…, *M*:

(6) $$\begin{array}{c} A_{i}=s\left(i\right)\times U\left(:,i\right)\times V{\left(:,i\right)}^{T}. \end{array}$$

Therefore the matrix *A*_*i*_ contains the *i*th component; the extraction process is

(7) $$\begin{array}{c} C_{i}=\left[\begin{array}{cc} A_{i}\left(1,:\right) & A_{i}{\left(2,N:M\right)}^{T} \end{array}\right], \end{array}$$

where *C*_*i*_ is the *i*th component and the elements of *C*_*i*_ are located in the first row and last column of *A*_*i*_.

The energy of the obtained components is computed with

(8) $$\begin{array}{c} E_{i}=\frac{s_{i}^{2}}{\sum_{i=1}^{M}s_{i}^{2}}, \end{array}$$

where *E*_*i*_ is the energy of the *i*th component and *s*_*i*_ is the *i*th singular value. When *M* > 2, the component *C*_*H*_ is computed with the sum of the components from 2 to *M*, as follows:

(9) $$\begin{array}{c} C_{H}=\overset{M}{\underset{i=2}{\sum }}C_{i}. \end{array}$$

## 3. Proposed Forecasting Models

### 3.1. Autoregressive Integrated Moving Average Model

The ARIMA model is the generalization of the ARMA model; ARIMA processes are applied on nonstationary time series to convert them in stationary, in ARIMA(*P*, *D*, *Q*) process; *D* is a nonnegative integer that determines the order and *P* and *Q* are the polynomials degrees [40].

The time series transformation process to obtain a stationary time series from a nonstationary is developed by means of differentiation; the time series *x*_*t*_ will be nonstationary of order *d* if *x*_*t*_ = Δ^*d*^ *x*_*t*_ is stationary; the transformation process is

(10a) $$\begin{array}{c} \Delta x_{t}=x_{t}-x_{t-1}, \end{array}$$

(10b) $$\begin{array}{c} \Delta^{j+1}x_{t}=\Delta^{j}x_{t}-\Delta^{j}x_{t-1}, \end{array}$$

where *x* is the time series, *t* is the time instant, and *j* is the number of differentiations obtained, that is, because the process is iterative. Once we obtained the stationary time series, the estimation is computed with

(11) $$\begin{array}{c} {\hat{x}}_{t}=\overset{P}{\underset{i=1}{\sum }}\alpha_{i}z_{t-i}+\overset{Q}{\underset{i=1}{\sum }}\beta_{i}e_{t-i}+e_{t}, \end{array}$$

where *α*_*i*_ represents the coefficients of the AR terms of order *P* and *β*_*i*_ denotes the coefficients of the MA terms of order *Q*, *z* is the input regressor vector, which is defined in Section 3.2, and *e* is a source of randomness and is called white noise. The coefficients *α*_*i*_ and *β*_*i*_ are estimated using the maximum likelihood estimation (MLE) algorithm [40].

### 3.2. Neural Network Forecasting Model

The ANN has a common structure of three layers [41]; the inputs are the lagged terms contained in the regressor vector *z*; at hidden layer the sigmoid transfer function is applied, and at output layer the forecasted value is obtained. The ANN output is

(12a) $$\begin{array}{c} \hat{x}\left(n\right)=\overset{Q}{\underset{j=1}{\sum }}v_{j}h_{j}, \end{array}$$

(12b) $$\begin{array}{c} h_{j}=f\left[\overset{K}{\underset{i=1}{\sum }}w_{ji}z_{i}\left(n\right)\right], \end{array}$$

where $\hat{x}$ is the estimated value, *n* is the time instant, *Q* is the number of hidden nodes, *v*_*j*_ and *w*_*ji*_ are the linear and nonlinear weights of the ANN connections, respectively, *z*_*i*_ represents the *i*th lagged term, and *f*(·) is the sigmoid transfer function denoted by

(13) $$\begin{array}{c} f\left(x\right)=\frac{1}{1+e^{-x}}. \end{array}$$

The lagged terms are the input of the ANN and they are contained in the regressor vector *z*, whose representation for MA smoothing is

(14) $$\begin{array}{c} Z\left(t\right)=\left[\tilde{s}\left(t-1\right),\tilde{s}\left(t-2\right),\ldots ,\tilde{s}\left(t-K\right)\right], \end{array}$$

where *K* = *P* lagged terms and *P* and *Q* were defined in Section 3.1.

The representation of *z* for HSVD smoothing is

(15) z(t)=[CL(t−1),…,CL(t−K),CH(t−1),…,CH(t−K)],

where *K* = 2*P* lagged terms.

The ANN is denoted by ANN(*K*, *Q*, 1), with *K* inputs, *Q* hidden nodes, and 1 output. The parameters *v* and *w* are updated with the application of two learning algorithms: one based on PSO and the other on RPROP.

#### 3.2.1. Learning Algorithm Based on PSO

The weight of the ANN connections, *w* and *v* are adjusted with PSO learning algorithm. In the swarm the *N*_*p*_ particles have a position vector *X*_*i*_ = (*X*_*i*1_, *X*_*i*2_,…, *X*_*iD*_) and a velocity vector *V*_*i*_ = (*V*_*i*1_, *V*_*i*2_,…, *V*_*iD*_); each particle is considered a potential solution in a *D*-dimensional search space. During each iteration the particles are accelerated toward the previous best position denoted by *p*_*id*_ and toward the global best position denoted by *p*_*gd*_. The swarm has *N*_*p*_ rows and *D* columns and it is initialized randomly; *D* is computed with *P* × *N*_*h*_ + *N*_*h*_; the process finishes when the lowest error is obtained based on the fitness function evaluation or when the maximum number of iterations is reached [42], as follows:

(16a) Vidl+1=Il×Vidl+c1×rd1(pidl+Xidl) +c2×rd2(pgdl+Xidl),

(16b) $$\begin{array}{c} X_{id}^{l+1}=X_{id}^{l}+V_{id}^{l+1}, \end{array}$$

(16c) $$\begin{array}{c} I^{l}=I_{\max}^{l}-\frac{I_{\max}^{l}-I_{\min}^{l}}{\text{ite}{\text{r}}_{\max}}\times l, \end{array}$$

where *i* = 1,…, *N*_*p*_, *d* = 1,…, *D*; *I* denotes the inertia weight; *c*_1_ and *c*_2_ are learning factors, *rd*_1_ and *rd*_2_ are positive random numbers in the range [0,1] under normal distribution, and *l* is the *l*th iteration. Inertia weight has linear decreasing, *I*_max⁡_ is the maximum value of inertia, *I*_min⁡_ is the lowest, and iter_max⁡_ is total of iterations.

The particle *X*_*id*_ represents the optimal solution, in this case the set of weights *w* and *v* for the ANN.

#### 3.2.2. Learning Algorithm Based on Resilient Backpropagation

RPROP is an efficient learning algorithm that performs a direct adaptation of the weight step based on local gradient information; it is considered a first-order method. The update rule depends only on the sign of the partial derivative of the arbitrary error regarding each weight of the ANN. The individual step size Δ_*ij*_ is computed for each weight using this rule [33], as follows:

(17) $$\begin{array}{c} \Delta_{ij}^{\left(t\right)}≔\{\begin{array}{cc} \eta^{+}\cdot \Delta_{ij}^{t-1} & \text{if}\;{\frac{\partial E}{\partial w_{ij}}}^{\left(t-1\right)}{\frac{\partial E}{\partial w_{ij}}}^{\left(t\right)}>0,\\ \eta^{-}\cdot \Delta_{ij}^{t-1} & \text{if}\;{\frac{\partial E}{\partial w_{ij}}}^{\left(t-1\right)}{\frac{\partial E}{\partial w_{ij}}}^{\left(t\right)}<0,\\ \Delta_{ij}^{t-1} & \text{else}, \end{array} \end{array}$$

where 0 < *η* ^−^ < 1 < *η* ^+^. If the partial derivative ∂*E*/∂*w*_*ij*_ has the same sign for consecutive steps, the step size is slightly increased by the factor *η* ^+^ in order to accelerate the convergence, whereas if it changes the sign, the step size is decreased by the factor *η* ^−^. Additionally in the case of a change in the sign, there should be no adaptation in the succeeding step; in the practice this can be done by setting ∂*E*/∂*w*_*ij*_ = 0 in the adaptation rule Δ_*ij*_. Finally the weight update and the adaptation are performed after the gradient information of all the weights is computed.

## 4. Forecasting Accuracy Metrics

The forecasting accuracy is evaluated with the metrics root mean squared error (RMSE), generalized cross validation (GCV), mean absolute percentage error (MAPE), and relative error (RE):

(18) $$\begin{array}{c} \;\text{RMSE}=\sqrt{\frac{1}{N_{v}}\overset{N_{v}}{\underset{i=1}{\sum }}{\left(x_{i}-{\hat{x}}_{i}\right)}^{2}},\\ \;\text{GCV}=\frac{\text{RMSE}}{{\left(1-K/N_{v}\right)}^{2}},\\ \;\text{MAPE}=\left[\frac{1}{N_{v}}\overset{N_{v}}{\underset{i=1}{\sum }}\left|\frac{\left(x_{i}-{\hat{x}}_{i}\right)}{x_{i}}\right|\right]\times 100,\\ \;\text{RE}=\overset{N_{v}}{\underset{i=1}{\sum }}\frac{\left(x_{i}-{\hat{x}}_{i}\right)}{x_{i}}, \end{array}$$

where *N*_*v*_ is the validation (testing) sample size, *x*_*i*_ is the *i*th observed value, ${\hat{x}}_{i}$ is the *i*th estimated value, and *K* is the length of the input regressor vector.

## 5. Results and Discussions

**Figure 2:**
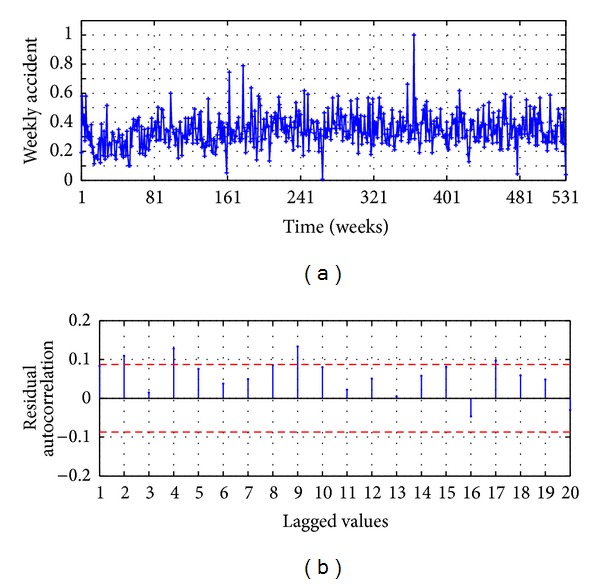
Accidents time series: (a) raw data and (b) autocorrelation function.

The data used for forecasting is the time series of injured people in traffic accidents occurring in Valparaíso, from 2003 to 2012; they were obtained from CONASET, Chile [43]. The data sampling period is weekly, with 531 registers as shown in Figure 2(a); the series was separated for training and testing, and by trial and error the 85% for training and the 15% for testing were determined.

### 5.1. ARIMA Forecasting

#### 5.1.1. Moving Average Smoothing

**Figure 3:**
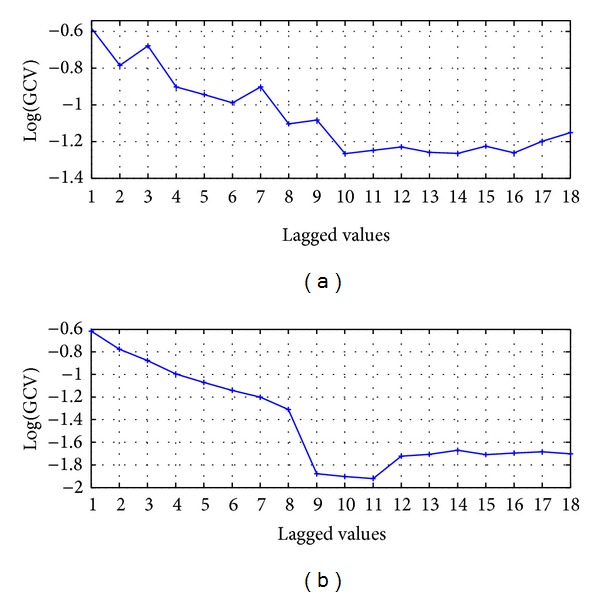
(a) MA smoothing and (b) HSVD smoothing.

The raw time series is smoothed using 3-point moving average, whose obtained values are used as input of the forecasting model ARIMA(*P*, *D*, *Q*); this is presented in Figure 1(a). The effective order of the polynomial for the AR terms is found to be *P* = 9 and the differentiation parameter is found to be *D* = 0; those values were obtained from the autocorrelation function (ACF) shown in Figure 2(b); to set the order *Q* of MA terms, is evaluated the metric GCV versus the *Q* Lagged values. The results of the GCV are presented in Figure 3(a); it shows that the lowest GCV is achieved with 10 lagged values. Therefore the configuration of the model is denoted by AM-ARIMA(9,0,10).

**Figure 4:**
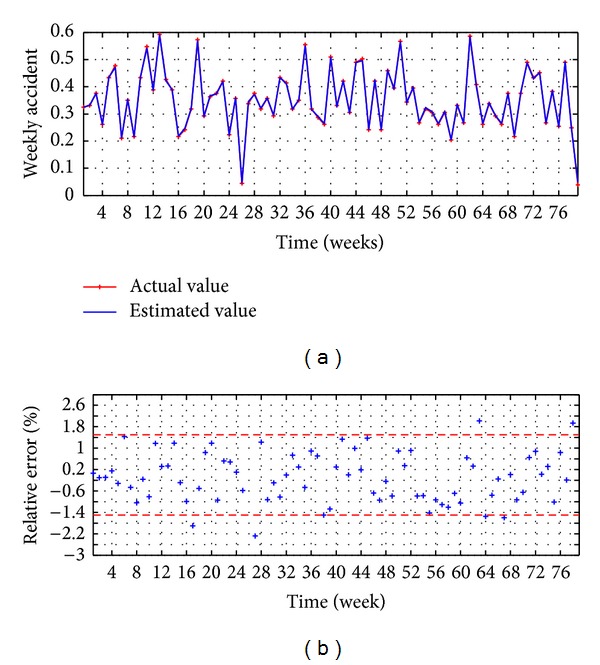
MA-ARIMA(9,0,10), (a) observed versus estimated (b) relative error.

**Figure 5:**
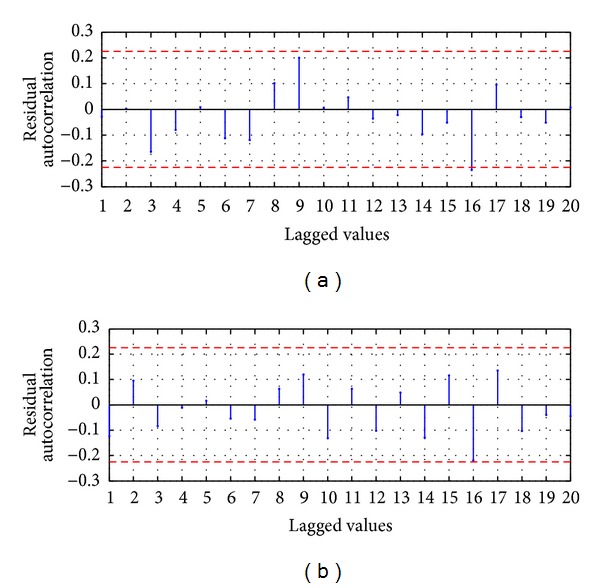
Residual ACF: (a) MA-ARIMA(9,0,10) and (b) SVD-ARIMA(9,0,11).

The evaluation executed in the testing stage is presented in Figures 4 and 5(a) and Table 1. The observed values versus the estimated values are illustrated in Figure 4(a), reaching a good accuracy, while the relative error is presented in Figure 4(b), which shows that the 87% of the points present an error lower than ±1.5%.

For the evaluation of the serial correlation of the model errors the ACF is applied, whose values are presented in Figure 5(a); it shows that ACF for a lag of 16 is slightly lower than the 95% confidence limit; however the rest of the coefficients are inside the confidence limit; therefore in the errors of the model AM-ARIMA(9,0,10) there is no serial correlation; we can conclude that the proposed model explains efficiently the variability of the process.

#### 5.1.2. HSVD Smoothing

**Figure 6:**
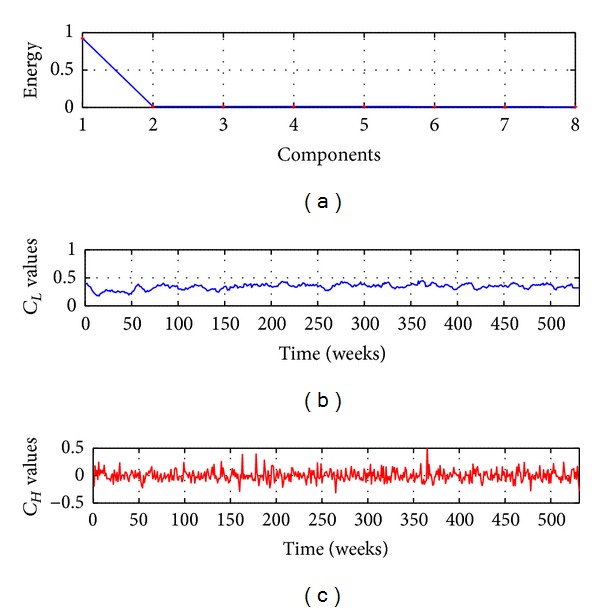
Accidents time series: (a) components energy, (b) low frequency component, and (c) high frequency component.

In this section the forecasting strategy presented in Figure 1(b) is evaluated; to implement this strategy in first instance the time series is mapped using the Hankel matrix, after the SVD process is executed to obtain the *M* components. The value of *M* is found through the computation of the singular values of the decomposition; this is presented in Figure 6(a); as shown in Figure 6(a), the major quantity of energy is captured by the two first components; therefore in this work only two components have been selected with *M* = 2. The first component extracted represents the long-term trend (*C*_*L*_) of the time series, while the second represents the short-term component of high frequency fluctuation (*C*_*H*_). The components *C*_*L*_ and *C*_*H*_ are shown in Figures 6(b) and 6(c), respectively.

To evaluate the model, in this section *P* = 9 and *D* = 0 are used, and *Q* is evaluated using the GCV metric for 1 ≤ *Q* ≤ 18; then the effective value *Q* = 11 is found, as shown in Figure 3(b); therefore the forecasting model is denoted by HSVD-ARIMA(9,0,11).

**Figure 7:**
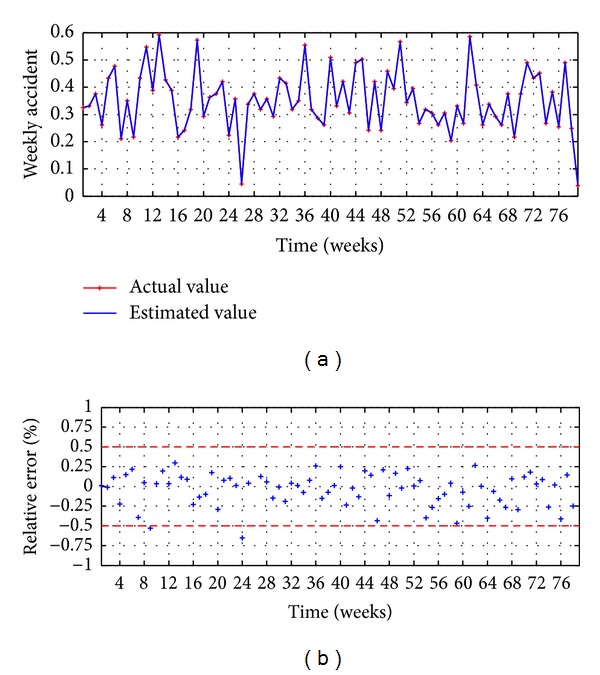
SVD-ARIMA(9,0,11): (a) observed versus estimated and (b) relative error.

Once *P* and *Q* are found, the forecasting is executed with the testing data set, and the results of HSVD-ARIMA(9,0,11) are shown in Figures 7(a), 7(b), and 5(b) and Table 1.  Figure 7(a) shows the observed values versus the estimates vales, and a good adjusting between them is found. The relative errors are presented in Figure 7(b); it shows that the 95% of the points present an error lower than ±0.5%.

For the evaluation of the serial correlation of the model errors the ACF is applied, whose values are presented in Figure 5(b); it shows that all the coefficients are inside the confidence limit; therefore in the model errors there is no serial correlation; we can conclude that the proposed model HSVD-ARIMA(9,0,11) explains efficiently the variability of the process.

The results presented in Table 1 show that the major accuracy is achieved with the model HSVD-ARIMA(9,0,11), with a RMSE of 0.00073 and a MAPE of 0.26%; the 95% of the points have a relative error lower than ±0.5%.

### 5.2. ANN Forecasting Model Based on PSO

#### 5.2.1. Moving Average Smoothing

The raw time series is smoothed using the moving average of order 3, whose obtained values are used as input of the forecasting model presented in Figure 1(a). The calibration executed in Section 5.1.1 is used for the neural network and then an ANN(*K*, *Q*, 1) is used, with *K* = 9 inputs (lagged values), *Q* = 10 hidden nodes, and 1 output.

**Figure 8:**
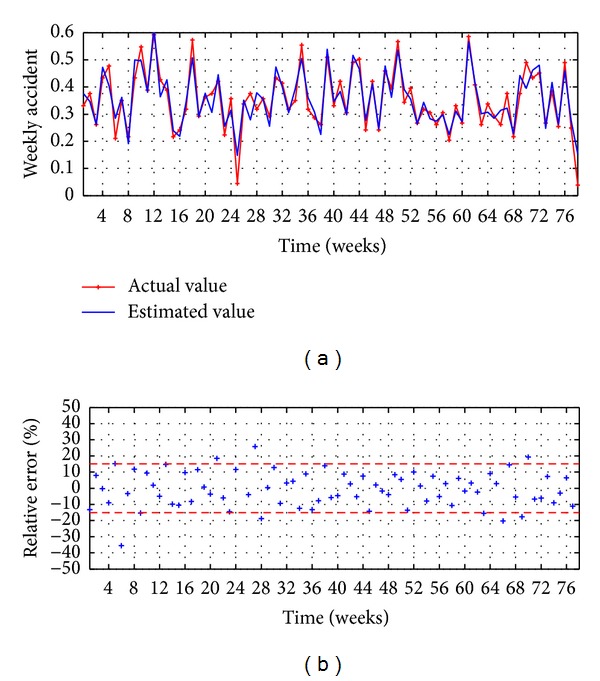
MA-ANN-PSO(9,10,1): (a) observed versus estimated and (b) relative error.

**Figure 9:**
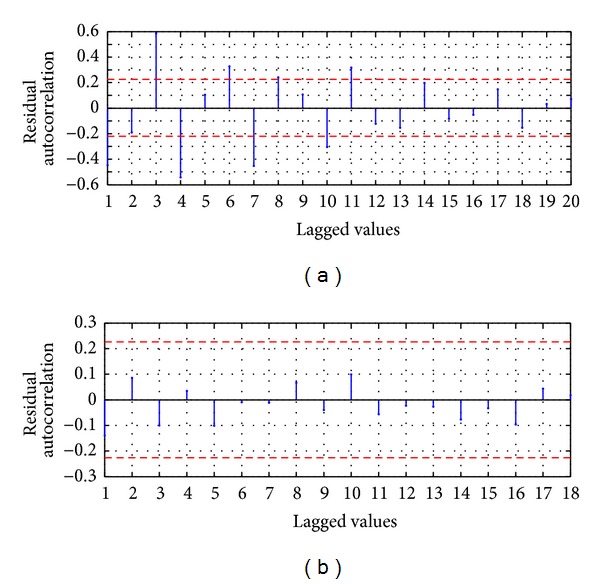
Residual ACF: (a) MA-ANN-PSO(9,10,1) and (b) HSVD-ANN-PSO(9,11,1).

The evaluation executed in the testing stage is presented in Figures 8 and 9(a) and Table 2. The observed values versus the estimated values are illustrated in Figure 8(a), reaching a good accuracy, while the relative error is presented in Figure 8(b), which shows that the 85% of the points present an error lower than ±15%.

For the evaluation of the serial correlation of the model errors the ACF is applied, whose values are presented in Figure 9(a); it shows that there are values with significative difference from zero to 95% of the confidence limit; by example the three major values are obtained when the lagged value is equal to 3, 4, and 7 weeks. Therefore in the residuals there is serial correlation; this implies that the model MA-ANN-PSO(9,10,1) is not recommended for future usage and probably other explanatory variables should be added in the model.

**Figure 10:**
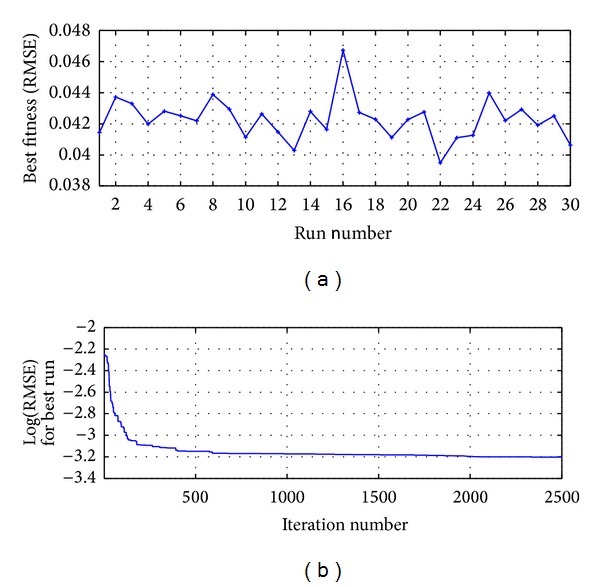
MA-ANN-PSO(9,10,1): (a) run versus fitness for 2500 iterations and (b) iterations number for the best run.

The process was run 30 times and the best result was reached in the run 22 as shown in Figure 10(a); Figure 10(b) presents the RMSE metric for the best run.

#### 5.2.2. HSVD Smoothing

In this section the forecasting strategy presented in Figure 1(b) is evaluated; the HSVD smoothing strategy is applied using the same calibration explained in Section 5.1.2; then an ANN(*K*, *Q*, 1) is used, with *K* = 9 inputs (lagged values), *Q* = 11 hidden nodes, and 1 output.

**Figure 11:**
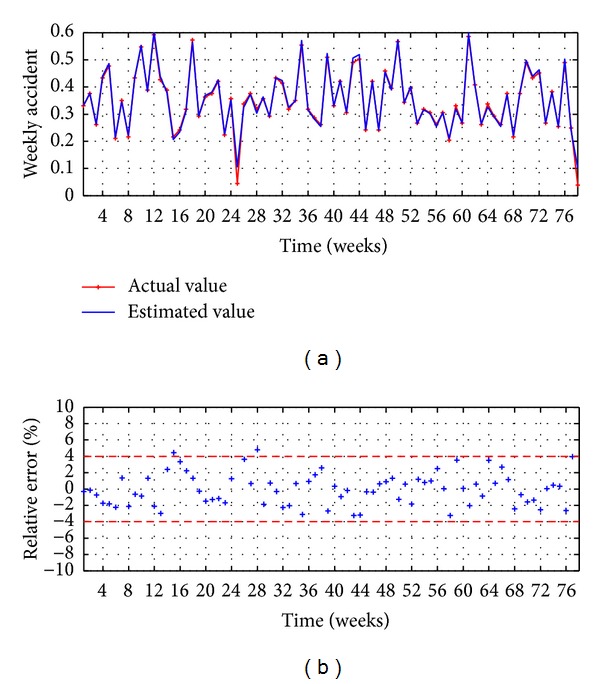
HSVD-ANN-PSO(9,11,1): (a) observed versus estimated and (b) relative rrror.

The evaluation executed in the testing stage is presented in Figures 11 and 9(b) and Table 2. The observed values versus the estimated values are illustrated in Figure 11(a), reaching a good accuracy, while the relative error is presented in Figure 11(b), which shows that the 95% of the points present an error lower than ±4%.

For the evaluation of the serial correlation of the model errors the ACF is applied, whose values are presented in Figure 9(b); it shows that all the coefficients are inside the confidence limit of 95% and statistically are equal to zero; therefore in the model errors there is no serial correlation; we can conclude that the proposed model HSVD-ANN-PSO(9,11,1) explains efficiently the variability of the process.

**Figure 12:**
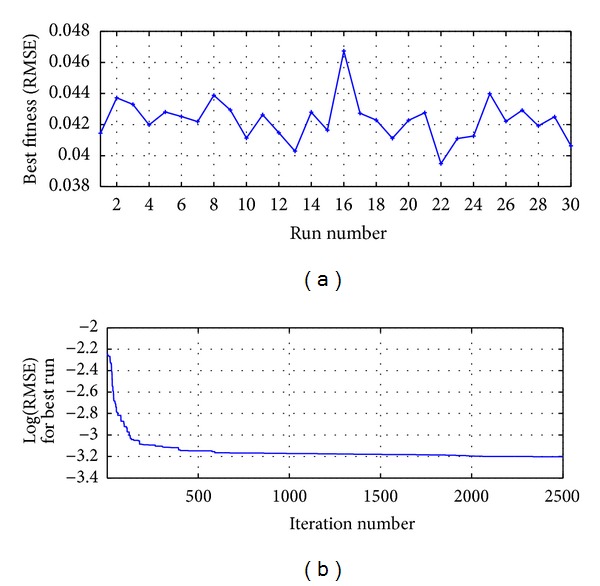
HSVD-ANN-PSO(9,11,1): (a) run versus fitness for 2500 iterations and (b) iterations number for the best run.

The process was run 30 times and the best result was reached in the run 11 as shown in Figure 12(a); Figure 12(b) presents the RMSE metric for the best run.

The results presented in Table 2 show that the major accuracy is achieved with the model HSVD-ANN-PSO(9,11,1), with a RMSE of 0.0123 and a MAPE of 5.45%; the 95% of the points have a relative error lower than ±4%.

### 5.3. ANN Forecasting Model Based on RPROP

#### 5.3.1. Moving Average Smoothing

The raw time series is smoothed using the moving average of order 3, whose obtained values are used as input of the forecasting model presented in Figure 1(a). The calibration executed in Section 5.1.1 is used for the neural network; then an ANN(*K*, *Q*, 1) is used, with *K* = 9 inputs (lagged values), *Q* = 10 hidden nodes, and 1 output.

**Figure 13:**
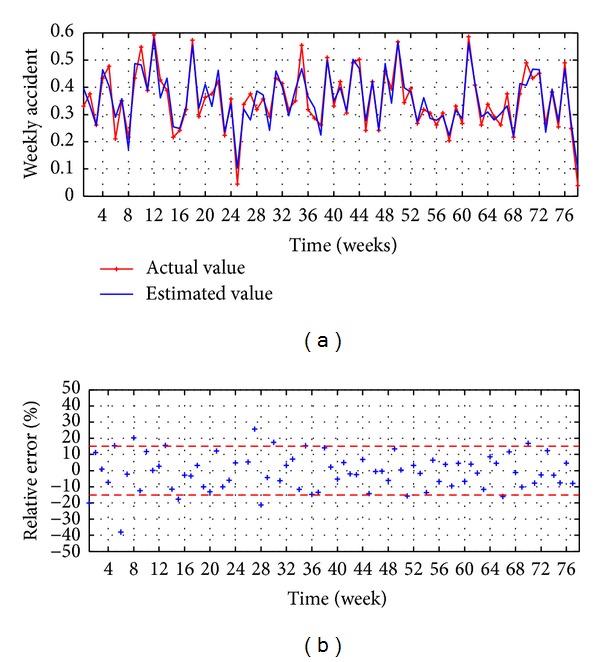
MA-ANN-RPROP(9,10,1): (a) observed versus estimated and (b) relative error.

**Figure 14:**
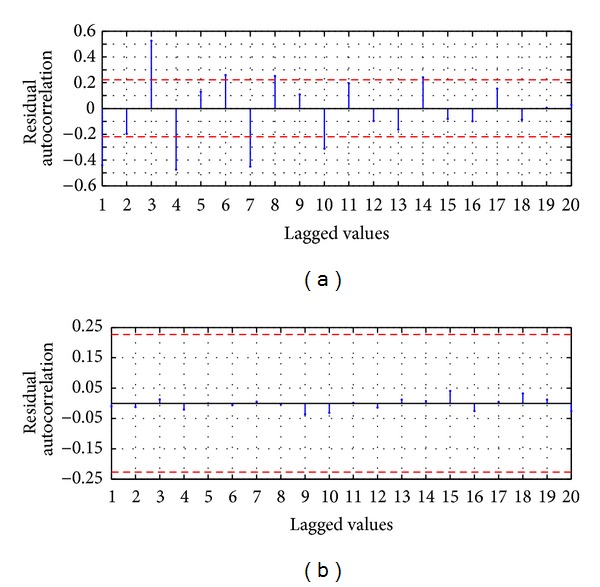
Residual ACF: (a) MA-ANN-RPROP(9,10,1) and (b) HSVD-ANN-RPROP(9,11,1).

The evaluation executed in the testing stage is presented in Figures 13 and 14(a) and Table 3. The observed values versus the estimated values are illustrated in Figure 13(a), reaching a good accuracy, while the relative error is presented in Figure 13(b), which shows that the 81% of the points present an error lower than ±15%.

For the evaluation of the serial correlation of the model errors the ACF is applied, whose values are presented in Figure 14(a); it shows that there are values with significative difference from zero to 95% of the confidence limit; by example the three major values are obtained when the lagged value is equal to 3, 4, and 7 weeks. Therefore in the residuals there is serial correlation; this implies that the model MA-ANN-RPROP(9,10,1) is not recommended for future usage and probably other explanatory variables should be added in the model.

**Figure 15:**
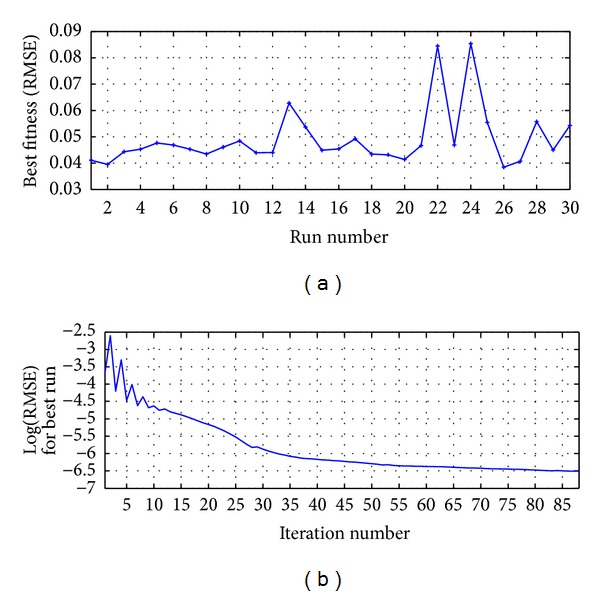
MA-ANN-RPROP(9,10,1): (a) run versus fitness for 85 iterations and (b) iterations number for the best run.

The process was run 30 times, and the best result was reached in the run 26 as shown in Figure 15(a); Figure 15(b) presents the RMSE metric for the best run.

#### 5.3.2. HSVD Smoothing

In this section the forecasting strategy presented in Figure 1(b) is evaluated, the HSVD smoothing strategy is applied using the same calibration explained in Section 5.1.2, and then an ANN(*K*, *Q*, 1) is used, with *K* = 9 inputs (lagged values), *Q* = 11 hidden nodes, and 1 output.

**Figure 16:**
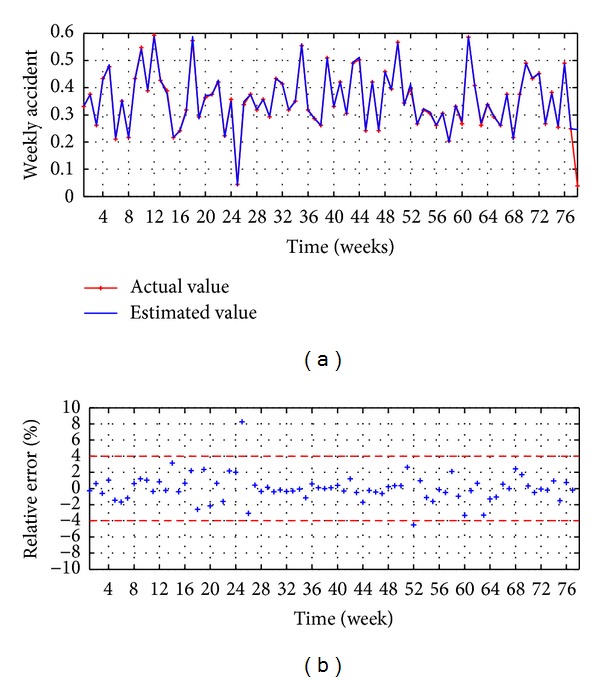
HSVD-ANN-RPROP(9,11,1): (a) observed versus estimated and (b) relative error.

The evaluation executed in the testing stage is presented in Figures 16 and 14(b) and Table 3. The observed values versus the estimated values are illustrated in Figure 16(a), reaching a good accuracy, while the relative error is presented in Figure 16(b), which shows that the 96% of the points present an error lower than ±4%.

**Figure 17:**
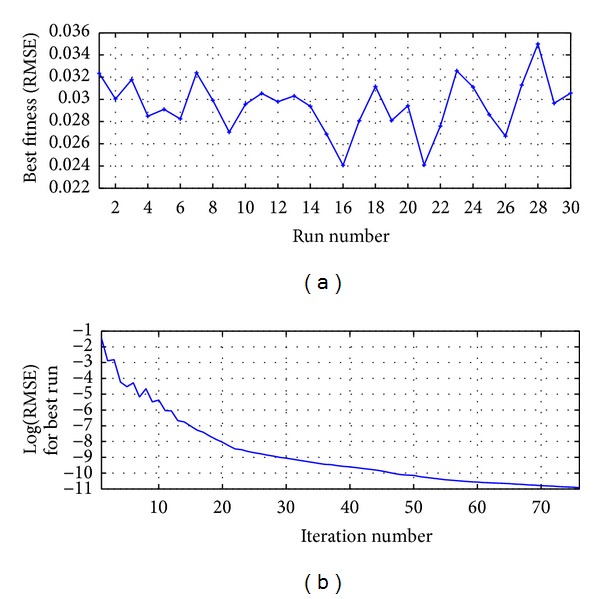
HSVD-ANN(9,11,1): (a) run versus fitness for 70 iterations and (b) iterations number for the best run.

For the evaluation of the serial correlation of the model errors the ACF is applied, whose values are presented in Figure 14(b); it shows that all the coefficients are inside the confidence limit and statistically are equal to zero; therefore in the model errors there is no serial correlation; we can conclude that the proposed model HSVD-ANN-RPROP(9,11,1) explains efficiently the variability of the process. The process was run 30 times and the first best result was reached in the run 21 as shown in Figure 17(a); Figure 17(b) presents the RMSE metric for the best run.

The results presented in Table 3 show that the major accuracy is achieved with the model HSVD-ANN-RPROP(9,11,1), with a RMSE of 0.024 and a MAPE of 8.08%; the 96% of the points have a relative error lower than ±4%.

Finally, Pitman's correlation test [44] is used to compare all forecasting models in a pairwise fashion. Pitman's test is equivalent to testing if the correlation (Corr) between *Υ* and Ψ is significantly different from zero, where *Υ* and Ψ are defined by

(19a) $$\begin{array}{c} \Upsilon =e_{1}\left(n\right)+e_{2}\left(n\right),\;n=1,2,\ldots ,N_{v}, \end{array}$$

(19b) $$\begin{array}{c} \Psi =e_{1}\left(n\right)-e_{2}\left(n\right),\;n=1,2,\ldots ,N_{v}, \end{array}$$

where *e*_1_ and *e*_2_ represent the one-step-ahead forecast error for model 1 and model 2, respectively. The null hypothesis is significant at the 5% significance level if $|\text{Corr}|>1.96/\sqrt{N_{v}}$.

The evaluated correlations between *Υ* and Ψ are presented in Table 4.

The results presented in Table 4 show that statistically there is a significant superiority of the HSVD-ARIMA forecasting model, regarding the rest of models. The results are presented from left to right, where the first is the best model and the last is the worst model.

## 6. Conclusions

In this paper were proposed two strategies of time series smoothing to improve the forecasting accuracy. The first smoothing strategy is based on moving average of order 3, while the second is based on the Hankel singular value decomposition. The strategies were evaluated with the time series of traffic accidents occurring in Valparaíso, Chile, from 2003 to 2012.

The estimation of the smoothed values was developed through three conventional models, ARIMA, an ANN based on PSO, and an ANN based on RPROP. The comparison of the six models implemented shows that the first best model is HSVD-ARIMA, as it obtained the major accuracy, with a MAPE of 0.26% and a RMSE of 0.00073, while the second best is the model MA-ARIMA, with a MAPE of 1.12% and a RMSE of 0.0034. On the other hand, the model with the lowest accuracy was MA-ANN-PSO with a MAPE of 15.51% and a RMSE of 0.041. Pitman's test was executed to evaluate the difference of the accuracy between the six proposed models and the results show that statistically there is a significant superiority of the forecasting model based on HSVD-ARIMA. Due to the high accuracy reached with the best model, in future works, it will be applied to evaluate new time series of other regions and countries.

## Figures and Tables

**Table 1:** Forecasting with ARIMA.

	MA-ARIMA	HSVD-ARIMA
RMSE	0.0034	0.00073
MAPE	1.12%	0.26%
GCV	0.006	0.0013
RE ± 1.5%	87%	—
RE ± 0.5%	—	95%

**Table 2:** Forecasting with ANN-PSO.

	MA-ANN-PSO	HSVD-ANN-PSO
RMSE	0.04145	0.0123
MAPE	15.51%	5.45%
GCV	0.053	0.022
RE ± 15%	85%	—
RE ± 4%	—	95%

**Table 3:** Forecasting with ANN-RPROP.

	MA-ANN-RPROP	HSVD-ANN-RPROP
RMSE	0.0384	0.024
MAPE	12.25%	8.08%
GCV	0.0695	0.045
RE ± 15%	81%	—
RE ± 4%	—	96%

**Table 4:** Pitman's correlation (Corr) for pairwise comparison six models at 5% of significance and the critical value 0.2219.

Models	M1	M2	M3	M4	M5	M6
M1 ≔ HSVD-ARIMA	—	−0.9146	−0.9931	−0.9983	−0.9994	−0.9993
M2 ≔ MA-ARIMA	—	—	−0.8676	−0.9648	−0.9895	−0.9887
M3 ≔ HSVD-ANN-PSO	—	—	—	−0.6645	−0.8521	−0.8216
M4 ≔ HSVD-ANN-RPRO	—	—	—	—	−0.5129	−0.4458
M5 ≔ ANN-RPROP	—	—	—	—	—	0.1623
M6 ≔ MA-ANN-PSO	—	—	—	—	—	—
